# Oral Administration of Probiotics Increases Paneth Cells and Intestinal Antimicrobial Activity

**DOI:** 10.3389/fmicb.2018.00736

**Published:** 2018-04-16

**Authors:** Silvia I. Cazorla, Carolina Maldonado-Galdeano, Ricardo Weill, Juan De Paula, Gabriela D. V. Perdigón

**Affiliations:** ^1^Laboratorio de Inmunología, Centro de Referencia para Lactobacilos (CERELA-CONICET), San Miguel de Tucumán, Argentina; ^2^Cátedra de Inmunología, Facultad de Bioquímica, Química y Farmacia, Universidad Nacional de Tucumán, San Miguel de Tucumán, Argentina; ^3^Departamento de Investigación y Desarrollo, DANONE, Buenos Aires, Argentina; ^4^Servicio de Gastroenterología, Hospital Italiano de Buenos Aires, Buenos Aires, Argentina

**Keywords:** probiotics, antimicrobial activity, Paneth cells, intestinal fluids, intestinal microbiota

## Abstract

The huge amount of intestinal bacteria represents a continuing threat to the intestinal barrier. To meet this challenge, gut epithelial cells produce antimicrobial peptides (AMP) that act at the forefront of innate immunity. We explore whether this antimicrobial activity and Paneth cells, the main intestinal cell responsible of AMP production, are influenced by probiotics administration, to avoid the imbalance of intestinal microbiota and preserve intestinal barrier. Administration of *Lactobacillus casei* CRL 431 (Lc 431) and *L. paracasei* CNCM I-1518 (Lp 1518) to 42 days old mice, increases the number of Paneth cells on small intestine, and the antimicrobial activity against the pathogens *Staphylococcus aureus* and *Salmonella* Typhimurium in the intestinal fluids. Specifically, strong damage of the bacterial cell with leakage of cytoplasmic content, and cellular fragmentation were observed in *S.* Typhimurium and *S. aureus*. Even more important, probiotics increase the antimicrobial activity of the intestinal fluids at the different ages, from weaning (21 days old) to old age (180 days old). Intestinal antimicrobial activity stimulated by oral probiotics, do not influence significantly the composition of total anaerobic bacteria, lactobacilli and enterobacteria in the large intestine, at any age analyzed. This result, together with the antimicrobial activity observed against the same probiotic bacteria; endorse the regular consumption of probiotics without adverse effect on the intestinal homeostasis in healthy individuals. We demonstrate that oral probiotics increase intestinal antimicrobial activity and Paneth cells in order to strengthen epithelial barrier against pathogens. This effect would be another important mechanism by which probiotics protect the host mainly against infectious diseases.

## Introduction

The epithelium of the intestine is the largest surface area exposed to the outer environment, including pathogens, toxins, and foods. More than 1 × 10^14^ symbiotic microorganisms live in the human intestinal lumen. Nevertheless, intestinal infection or translocation of bacterial agents is an exception rather than the rule and is mostly limited to highly pathogenic bacteria or predisposing disease states. In order to face this challenge, mammalian intestine minimizes contact between luminal microorganisms and intestinal epithelial cells surface, through the development of key elements: mucus, IgA, and antimicrobial peptides (AMPs) (Hooper and Macpherson, 2010). Paneth cells, a characteristic epithelial cell of the small intestine located at the bottom of the intestinal crypts, are responsible for the secretion of a diverse arsenal of AMPs like lysozyme, secretory phospholipase A2 (sPLA2), defensins, defensin-like peptides (elafin and SLPI) and cathelicidins, which have a high antimicrobial activity.

Antimicrobial peptides are small molecules, generally smaller than 40 amino acids (aa), whose aa composition, net charge (generally cationic), as well as their amphipathic and size characteristics, favor their interaction with lipid bilayers, mainly those that form the cytoplasmic membranes of pathogens like fungi and protozoa (Nakamura et al., 2016).

Difference in the design of the membranes of microbes and multicellular animals’ allow AMPs to specifically target microorganisms. Bacterial membranes are organized in a way that the outermost leaflet of the bilayer is heavily populated by lipids with negatively charged phospholipids head groups. In contrast, the outer leaflet of eukaryotic’s membranes is composed principally by lipids without net charge; most of the lipids with negatively charged head groups are segregated into the inner leaflet, facing the cytoplasm (Zasloff, 2002; Sankaran-Walters et al., 2017).

The expression, secretion, and activity of epithelial AMPs are tightly controlled through multiple transcriptional and post-transcriptional mechanisms (Mukherjee and Hooper, 2015). Studies on germ free mice have demonstrated that some intestinal AMPs are expressed independently of the microbiota signals (Putsep et al., 2000). While others, stored in secretory granules, are released into the intestinal lumen on stimulation with microbe-associated molecular patterms (MAMPs) and inflammatory cytokines [e.g., interleukin (IL)-1β, tumor necrosis factor alpha (TNF-α) and IL-22] (Ayabe et al., 2000; Habil et al., 2014).

Several reports offered concrete insight into the multiple functions of AMPs from Paneth cell to protect against pathogen colonization and controlling microbiota composition and localization. Namely, (i) mice deficient in matrix metalloproteinase 7 (MMP-7), the Paneth cell α-defensin-processing enzyme, are highly susceptible to bacterial pathogens (Wilson et al., 1999); (ii) transgenic mice overexpressing the α- human defensin 5 (HD5) are protected against an oral challenge with *Salmonella* Typhimurium, with a significant increase in their survival (Salzman et al., 2003); (iii) *in vivo* depletion or down regulation of RegIII-γ, markedly decreases the killing of *Listeria monocytogenes* and vancomycin-resistant Enterococcus (Brandl et al., 2007, 2008); (iv) in both MMP-7 deficient and in α-defensin 5 (DEFA5)+/+ mice, the microbiota composition in the small intestine was dramatically different from that of the wild-type strain (Salzman, 2010); and (v) genetic defects associated to decreased AMPs production can be related to intestinal inflammation and disease (Wehkamp et al., 2005; Cadwell et al., 2008; Koslowski et al., 2010). In addition to their antimicrobial role, AMPs have been shown to act as chemoattractants for cells of innate and adaptive immunity, and so, many authors consider them as a bridge between innate and adaptive immunity (Steinstraesser et al., 2011; Rivas-Santiago et al., 2013).

In the last century, probiotics have gained more and more interest as alternatives to stimulate the immune system, restricting the use of antibiotics or anti-inflammatory drugs. Probiotics are defined by the FAO/WHO as live microorganisms which, when administered in adequate amounts, confer a health benefit on the host. There are several benefits set for probiotics: protection against infections (Acurcio et al., 2017; Mallina et al., 2017; Park et al., 2017), prevention of cancer (Aragón et al., 2015), regulation of peristalsis, reduction of symptoms associated with lactose intolerance (Pakdaman et al., 2016), decrease in gut inflammatory response (Fábrega et al., 2017) and prevention of allergy (Vélez et al., 2015; Nelson, 2016). Most of these profits have been associated to the modulation that probiotics or fermented milk elicit in innate as well as the acquired immune system (Maldonado Galdeano et al., 2015; Igarashi et al., 2017).

In the present work we explore in a murine model whether Paneth cells and the antimicrobial intestinal activity are influenced by probiotics oral administration, in order to kill pathogens and strengthen the intestinal barrier integrity. Additionally, we investigate probiotics effects along the lives of the mice on AMP production, mainly on aging animals.

## Materials and Methods

### Bacteria

*Lactobacillus casei* CRL 431 (Morata de Ambrosini et al., 1999) and *L. fermentum* CRL 1386 (Vinderola et al., 2004) were obtained from the CERELA Culture Collection (San Miguel de Tucumán, Argentina). *L. paracasei* CNCM I-1518 was provided for DANONE Argentina. These strains were cultured for 16 h at 37°C in a sterile Man-Rogosa-Sharpe (MRS) broth (Britania, Buenos Aires, Argentina).

*Staphylococcus aureus* ATCC 25923 were kindly provided by Mariel Cáceres, Hospital Ángel C. Padilla, Tucumán. *Salmonella enterica serovar* Typhimurium strain was obtained from the Bacteriology Department of the Hospital del Niño Jesus (San Miguel de Tucumán, Argentina).

Aliquots of *S.* Typhimurium and *S. aureus* (200 μl) from an overnight culture were placed in 5 ml of sterile Brain Heart Infusion (BHI) broth (Britania, Buenos Aires, Argentina) and incubated during 4–5 h to reach exponential grown phase.

### Animals and Diet Supplementation

BALB/c mice were provided for CERELA (San Miguel de Tucumán, Argentina) from a closed random bred colony, and maintained in a room with a 12-h light/dark cycle at 22 ± 2°C, feeding *ad libitum* with conventional balanced food commercial. Mice of each control and test groups were sacrificed by cervical dislocation, and then small and large intestine and intestinal fluids from each mouse were removed for further studies. All animal protocols were preapproved by the Animal Protection Committee of CERELA (CRL-BIOT-LI-2017/3A) and conducted in accordance with the guidelines established by the Consejo Nacional de Investigaciones Científicas y Técnicas (CONICET) and the NHI^1,2^ .

Lactobacillus bacteria overnight cultures were grown at 37°C in 5 ml sterile Mann-Rogosa-Sharp (MRS) broth (Britania, Buenos Aires, Argentina). The cells were harvested by centrifugation at 5000 g for 10 min, washed three times with phosphate saline solution (PBS) and resuspended in 5 ml of sterile 10% (wt/vol) non-fat milk. Bacterial suspensions were diluted 1:30 in water and administered *ad libitum* to the mice. The final concentration of probiotics bacteria was 2 ± 1 × 10^8^ CFU/ml, a concentration extensively used by our group (Galdeano and Perdigón, 2004; Castillo et al., 2011). These counts were periodically controlled at the beginning of the administration and each 24 h of dilution in water to avoid modifications of more than one logarithmic unit. The probiotics bacteria were given in 5 ml of sterile 10% (wt/vol) skim milk powder and resuspended in the drinking water. The volume drank, controlled daily, was 2.5 to 3 ml/animal/day in both experimental and control animals as was reported previously (Galdeano and Perdigón, 2006; Balcells et al., 2017).

Groups of BALB/c mice from 21, 28, 35, 42, 54, 61, and 180 days old, received *L. casei* CRL 431 (Lc 431), *L. paracasei* CNCM I-1518 (Lp 1518), upon 7 and 5 days, respectively. These are the time required for an optimal activation of the intestinal immune system for each strain (Galdeano and Perdigón, 2006; de Moreno de LeBlanc et al., 2008; Vélez et al., 2015). Controls included mice that received indigenous Lactobacilli: *L. fermentum* CRL 1386, or non-Lactobacilli supplementation (conventional diet).

### Histological Samples

The small intestine samples were fixed in PBS-formaldehyde solution 10%, pH 7. The organs were rinsed with PBS and fixed for 24 h in 10% buffered formalin. After fixation, the tissues were dehydrated and embedded in paraffin, sectioned, stained with hematoxylin-eosin, and examined by light microscopy. A blind histological test of small intestine was done, analyzing 100 intestinal crypts in 5 slices of each organ.

HE stain allows the identification of Paneth cells (PCs) based on their distinctive granule staining pattern.

### Analysis of the Large Intestine Microbiota

Large intestines from the different mice were aseptically removed, weighed and placed in sterile tubes containing 5 ml of peptone water (0.1%). The samples were homogenized immediately under sterile conditions, using a microhomogenizer (MSE, England). Serial dilutions of the homogenized samples were performed and aliquots (0.1 ml) of dilutions were spread onto the surface of the following agarized media: Reinforced Clostridial Agar (RCA, Britania, Buenos Aires, Argentina) for total anaerobic bacteria, Mann-Rogosa-Sharp (MRS Britania, Buenos Aires, Argentina) for total lactobacilli (Britania, Buenos Aires, Argentina), and MacConkey for total enterobacteria. MacConkey and MRS agar were aerobically incubated at 37°C for 24 and 48 h, respectively. The other culture media were anaerobically incubated at 37°C for 72–96 h.

### *Ex Vivo* Analyses of the Antimicrobial Activity

According to previous reports showing that high ionic strength of bacterial broths is a major inactivation factor for the microbicidal activity of α-defensins and to match the low ionic strength and pH of the intestinal mucous layer, we used 10 mM sodium phosphate buffer, pH 7.4, in sterile distilled water. This medium does not compromise bacterial growth, as previously reported (Shimoda et al., 1995; Nakajima et al., 2003).

After animals were sacrificed, the small intestines were removed, and their content was collected in sterile tube by passage 0.5 ml of 10 mM sodium phosphate buffer, pH 7.4 along the intestine. Supernatant were then collected after centrifuge at 1300 × *g* 4°C 15 min. Exponential growth phase suspensions of *S.* Typhimurium, *S. aureus, L. casei* CRL 431, and *L. paracasei* CNCM I-1518 bacteria were washed twice in 10 mM sodium phosphate buffer pH 7.4, adjusted at 5 × 10^6^ CFU in 20 μl and incubated for 2 h at 37°C in the presence or absence of 100 μl of the intestinal fluids obtained from the different mice. Then, each incubation mixtures were serially diluted, spread in duplicate selective agar plates, and incubated at 37°C for 18 h, followed by determination of CFU count (Furci et al., 2015). Each assay was repeated three times independently. Results were expressed as the differences in the CFU/ml presents before and after the incubation of the bacteria with the intestinal fluids.

### Transmission Electron Microscopy Studies

Approximately 2 × 10^8^ CFU of *S.* Typhimurium, *S. aureus, L. casei* CRL 431, and *L. paracasei* CNCM I-1518 were washed twice in 10 mM sodium phosphate buffer, pH 7.4, and incubated for 1 h at 37°C in the presence or absence of 100 μl of the intestinal fluids from the different mice. As positive control, bacteria were incubated in the presence of 20 μg/ml of gentamicin.

The bacteria were then centrifuged at 5,000 *g* for 10 min, immersed phosphate-buffered glutaraldehyd and send to the “Centro Integral de Microscopía Electrónica (CIME) INSIBIO-UNT-CONICET,” for transmission electron microscopy analysis.

### Statistical Analyses

Results are presented as means ± SEM GraphPad Prism 5.0 software (GraphPad Software Inc., San Diego, CA, United States) was employed to carry out calculations. Results presented are representative of three independent experiments. The statistical significance was determined by one-way analysis of variance (ANOVA), using Kruskal–Wallis test performed with the GraphPad Prism 5.0 software. Comparisons were referred to the mice that received conventional diet. *p*-values < 0.05 were considered significant.

## Results

### Probiotics Oral Administration Increase the Numbers of Paneth Cells at the Base of the Intestine Crypt

**FIGURE 1:**
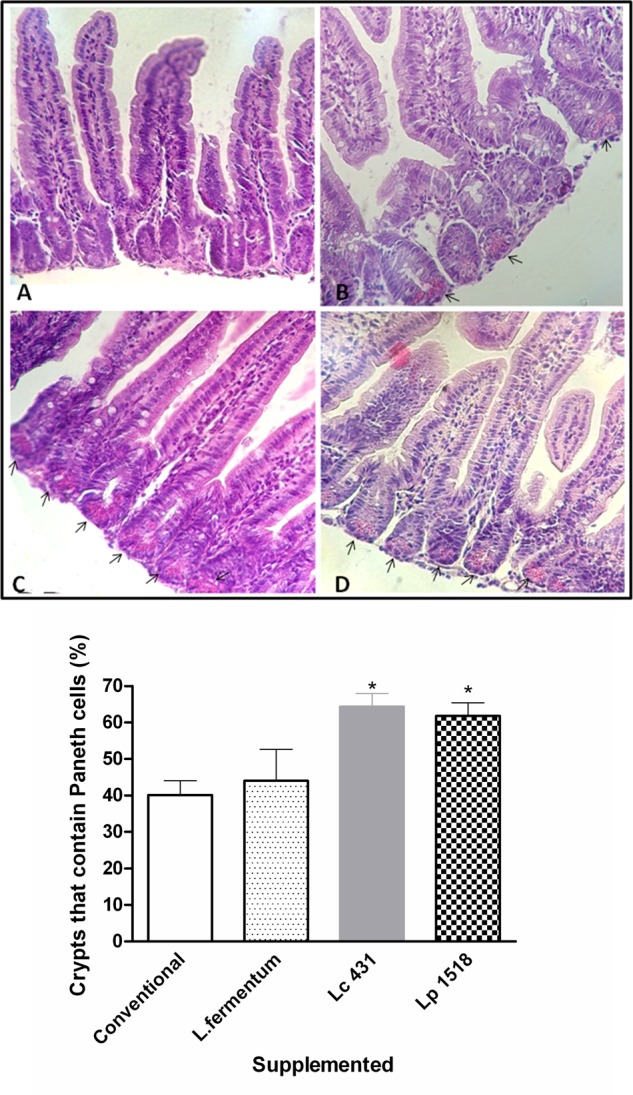
Paneth cells at the base of intestine crypt. Tissue sections of 42 days mice fed with conventional diet **(A)**, *Lactobacillus fermentum* **(B)**, *L. casei* CRL 431 **(C)**, or *L. paracasei* CNCM-I 1518 **(D)**, were stained with hematoxylin and eosin and examined by light microscopy. A blind histological test of small intestine was done by analyzing 100 intestinal crypts in 5 slices of each organ; and the percentages of crypts with positive Paneth cells were determined in each group. Photographs are representative of three independent experiments. Magnification 400×. Arrows point to the Paneth cells in the base of the crypt. The columns bar figure shows semi-quantitatively evaluation for each group. Each bar represents the group mean ± SEM. Results are representative of three independent experiments. ^∗^*p* < 0.05.

Forty two day old Balb/c mice were fed with: Group I: Conventional diet, Group II: *L. fermentum* as an intestinal commensal bacterium, Group III: *L. casei* CRL 431 (Lc 431), and Group IV: *L. paracasei* CNCM I-1518 (Lp 1518), for 5 and 7 days, respectively. At the end of this supplementation, animals were sacrificed and a hematoxylin and eosin stain was done in the small intestine tissue sections. We found that mice fed with any of the two probiotics bacteria (*L. casei* CRL 431 or *L. paracasei* CNCM I-1518), show a significant increase in the number of crypts with positive Paneth cells in the small intestine (64.29 ± 3.58 and 61.80 ± 5.31%, respectively), regarding to animals that received conventional diet (40.10 ± 3.9%) (Mean ± SEM). By contrast, animals fed with a bacteria that is part from the commensal intestinal microbiota, *L. fermentum*, show values similar to those that received conventional diet (44.00 ± 8.62%) (**Figure 1**).

**FIGURE 2:**
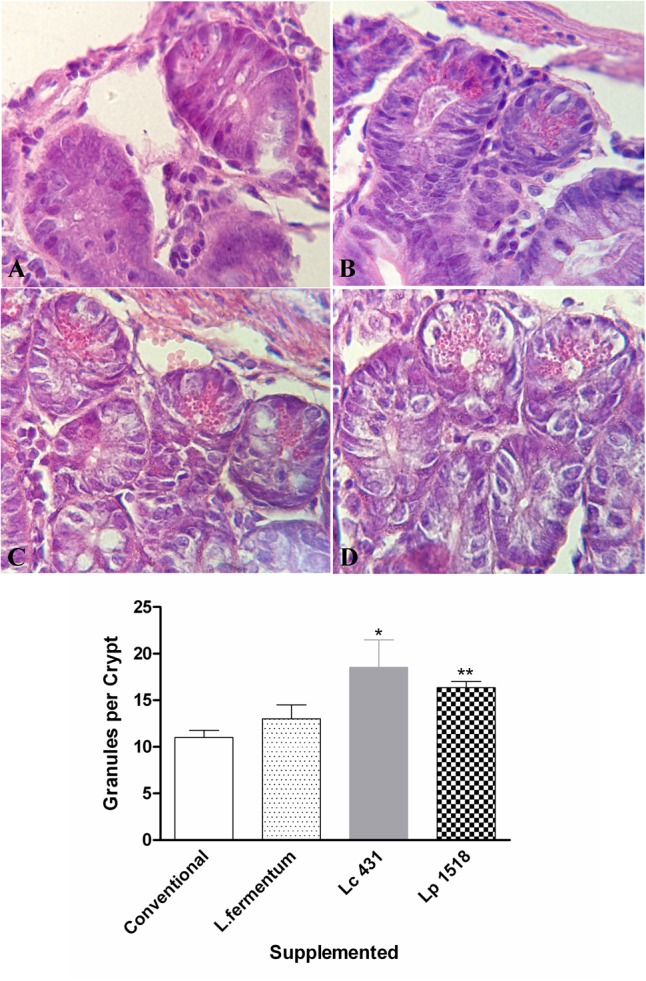
Micrographs of small intestine sections in the different animals’ models. Tissue sections of 42 days old mice fed with conventional diet **(A)**, *L. fermentum* **(B)**, *L. casei* CRL 431 **(C)**, or *L. paracasei* CNCM-I 1518 **(D)** stained with hematoxylin and eosin. Magnification 1000×. Results (mean ± SEM) are representative of three independent experiments. The differences were calculated with respect to the control group that received conventional diet ^∗^*p* < 0.05, ^∗∗^*p* < 0.01.

Additionally, we observed that mice supplemented with *L. casei* CRL 431, and *L. paracasei* CNCM I-1518 presented a significant increase in the number of granules per crypt with respect to those that received a conventional diet (*p* < 0.05, and *p* < 0.01, respectively) or *L. fermentum* (**Figure 2**).

### Probiotics Oral Supplementation Increases the Antimicrobial Activity in Intestinal Fluids

The intestinal fluids from adult’s mice (42 days old) fed for 7 or 5 days with the different diet were collected, and their antimicrobial activities against pathogen microorganism were studied *in vitro*. Since supplementation with *L. fermentum* did not induce a significant increase in the Paneth cells numbers (**Figures 1, 2**), this group was not included in the following studies.

**FIGURE 3:**
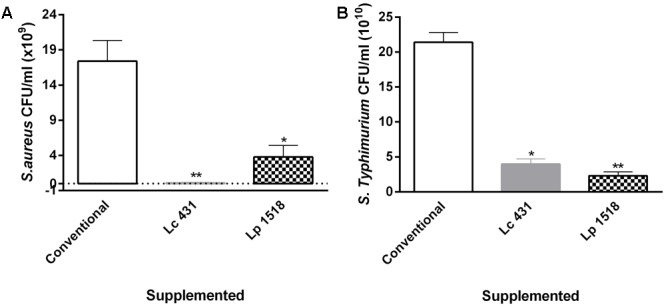
Antimicrobial activity of the animals’ intestinal fluids. *S. aureus* **(A)** and *S.* Typhimurium **(B)** (10^9^ CFU/ml) were incubated for 2 h at 37°C, in the presence of the intestinal fluids of 42 days mice fed with conventional diet, *L. casei* CRL 431 or *L. paracasei* CNCM-I 1518, for 7 and 5 days, respectively. After the co-incubation, viable bacteria were determined by plate count agar. A set of serial dilutions has been made and samples of each appropriate dilution were spread on top of solidified agar petri plates. Results are expressed as CFU/ml. Results (mean ± SEM) are representative of three independent experiments. Results were expressed as the differences in the CFU/ml before and after the incubation of the bacteria with the intestinal fluids. The statistics differences were calculated with respect to the control group that received conventional diet ^∗^*p* < 0.05, ^∗∗^*p* < 0.01.

We observed an important decrease in the CFU of *S. aureus* and *S.* Typhimurium after their incubation with the intestinal fluids of mice fed with *L. casei* CRL 431, compared with those of control mice intestinal fluids (*p* < 0.01 and *p* < 0.05, respectively). Similar results were found by the supplementation with *L. paracasei* CNCM-I 1518, in which a reduction of ∼4 and 10 times for *S. aureus* and *S.* Typhimurium, respectively, was observed, with respect to those fed with a conventional diet (**Figure 3**).

**FIGURE 4:**
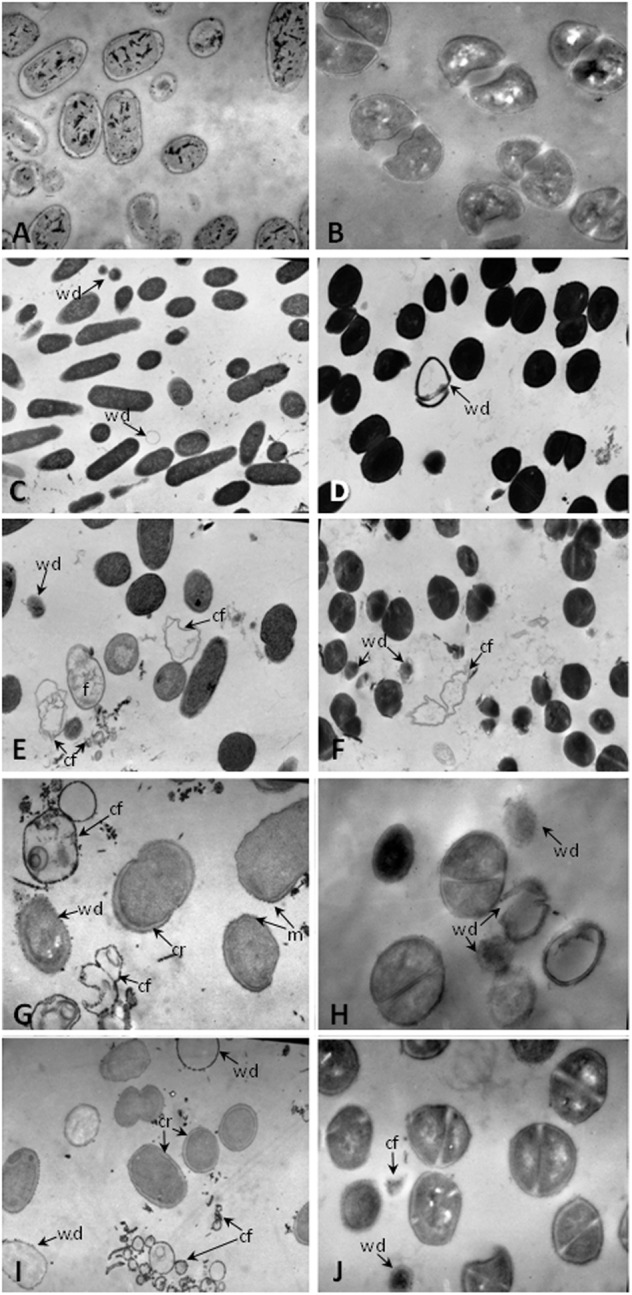
Transmission electron microscopy of pathogens undergoing to intestinal fluids of animals. Approximately 2 × 10^8^ CFU of **(A,C,E,G,I)** *S.* Typhimurium, **(B,D,F,H,J)** *S. aureus* were incubated for 1 h at 37°C in the presence of: **(A,B)** 10 mM sodium phosphate; the intestinal fluids of mice fed with: **(C,D)** conventional diet, **(E,F)** *L. paracasei* CNCM-I 1518, **(G–I)** *L. casei* CRL 431; and **(J)** Gentamicin 20 μg/ml. Magnification **(C)** 7500×; **(A,D–F,I)** 12.800×; **(B,G,H,J)** 22.800×. Cr, cytoplasmic retraction; f, electron-dense fibers; cf, cell fragmentation; wd, cell walls disruption; and m, membrane ruffling.

Furthermore, to gain insight into the mechanism by which the intestinal fluids led to the death of the pathogens, transmission electron microscopy was performed on thin sections of bacteria. Control bacteria exposed only to sodium phosphate buffer showed a regular cell shape with intact cell membranes and a homogenous cytoplasm (**Figures 4A,B**). By contrast, *S.* Typhimurium and *S. aureus* treated with the intestinal fluids of mice fed with Lc 431 or Lp 1518 revealed severe disruption of the wall cells with the later loss of internal cell contents and cell fragmentation. **Figure 4** also shows some cell membranes that appeared completely empty of cell contents, and cell debris appeared. We can also observe that the cytoplasm of cells exposed to the intestinal fluids of mice fed with *L. paracasei* CNCM I 1518, contained electron-dense fibers interspersed with electron translucent areas. Besides, bacteria exposed to the intestinal fluids of mice supplemented with the *L. casei* CRL 431 showed retractions of the intracellular and membrane ruffling (**Figures 4G–I**). It is important to mention, that bacteria incubated with the intestinal fluids of mice fed with a conventional diet revealed a slight cellular damage (**Figures 4C,D**). In this assay, we also include one positive control of cell damage. *S. aureus* was incubated with gentamicin, which irreversibly bind to specific 30S-subunit proteins and 16S rRNA, interrupting protein synthesis and causing the death of the bacteria (**Figure 4J**).

These results together mirror a strong activation on the antibacterial activity of the intestinal fluids upon supplementation with probiotic lactic bacteria. This antimicrobial activity could be explained by the presence of AMPs in the intestinal fluids, and the attachment of these cationic peptides to the negatively charged bacterial cell surface with the resulting depolarization and cell lysis.

**FIGURE 5:**
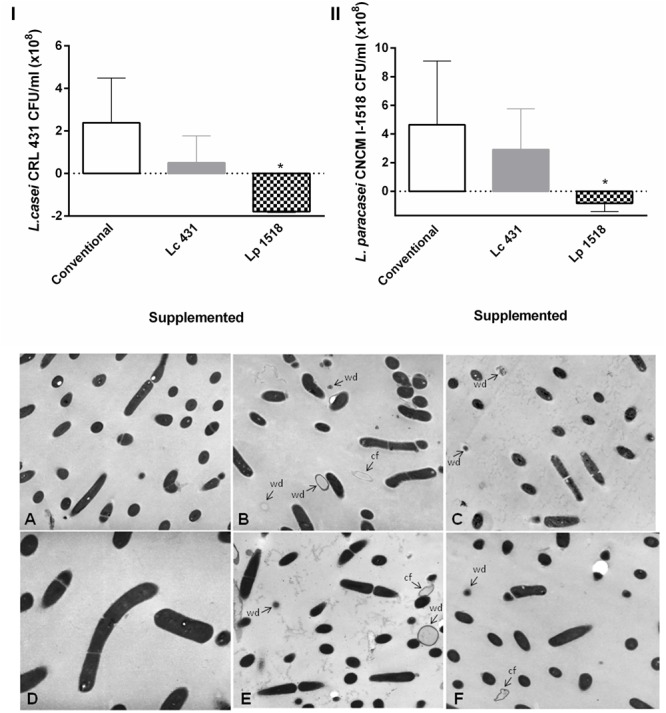
Effect of the animals’ intestinal fluids against the probiotics bacteria. In the upper panel *L. casei* CRL 431 **(I)** and *L. paracasei* CNCM-I 1518 **(II)** (10^8^ CFU/ml) were incubated for 2 h at 37°C, in the presence of the intestinal fluids of 42 days mice fed with Conventional diet, *L. casei* CRL431 or *L. paracasei* CNCM-I 1518. After the co-incubation, viable bacteria were determined by plate count agar. Results were expressed as the differences in the CFU/ml before and after the incubation of the bacteria with the intestinal fluids. The bars represent the average of 6 determinations, and the SEM is indicated by vertical lines. ^∗^*p* < 0.05. In the panel below, transmission electron microscopy studies were performed in **(A–C)** *L. casei* CRL 431 and **(D–F)** *L. paracasei* CNCM-I 1518, incubated for 1 h at 37°C in the presence of the intestinal fluids of mice fed as indicated: Conventional diet **(A,D)**, *L. casei* CRL 431 **(B,E)**, and *L. paracasei* CNCM-I 1518 **(C,F)**. Magnification (**A–C,E,F**: 12.800×), (**D**: 7.500×). cf, cell fragmentation; wd, cell walls disruption.

We further analyzed the effect of the intestinal fluids against the probiotics microorganisms, and observed that the intestinal fluids of mice fed with both probiotics had antibacterial activity against themselves: *L. casei* CRL431 and *L. paracasei* CNCM-I 1518. This activity was determined by bacterial plate counts and transmission microscopy (**Figure 5**). Interestingly, these last results highlight the relevance of the antibacterial activity present in the intestinal fluids, not only to protect the villus–crypt microenvironment as the portal of pathogens, but also to maintain the homeostasis in the intestinal microenvironment, avowing the internalization of the whole probiotic bacteria into the intestinal epithelial cells.

### The Antimicrobial Activity Induced by Oral Probiotics Is Also Effective in Aging Mice

**FIGURE 6:**
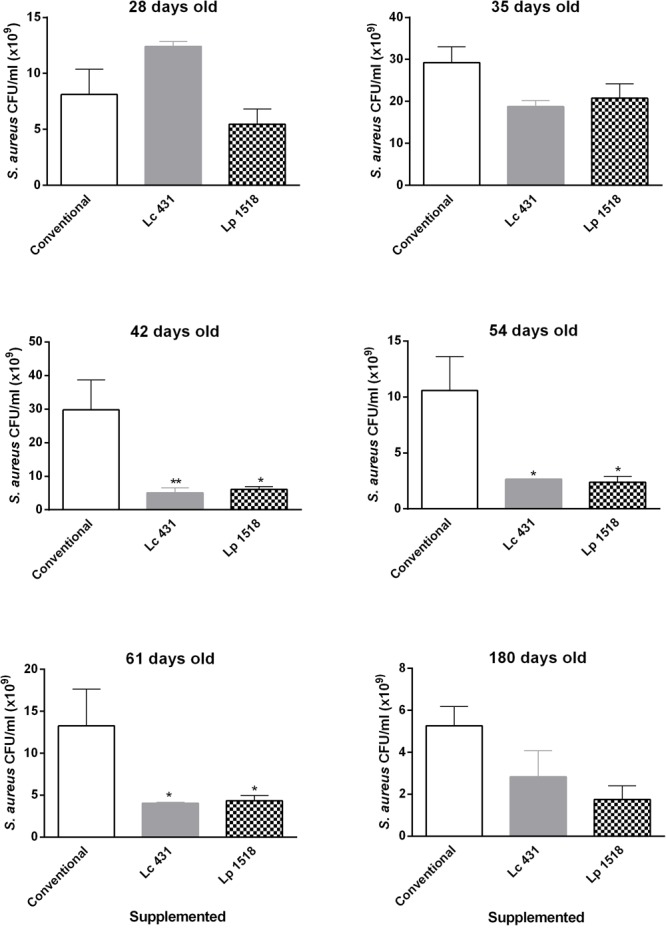
Antibacterial activity of the intestinal fluids throughout the lives of the animals. Twenty one, 28, 35, 42, 54, 61, and 180 days old mice were fed with conventional diet, *L. casei* CRL431 or *L. paracasei* CNCM-I 1518, for 7 and 5 days, respectively. At this time samples of intestinal fluids were taken and studied for their *S. aureus* antimicrobial activity after 2 h of incubation, by plate count agar. Results were expressed as the differences in the CFU/ml before and after the incubation of the bacteria with the intestinal fluids. Results expressed as CFU/ml (mean ± SEM) are representative of three independent experiments. The statistics differences were calculated with respect to the control group that received conventional diet ^∗^*p* < 0.05, ^∗∗^*p* < 0.01.

Later on, we analyzed the antibacterial activity according to the age of the animals, in the intestinal fluids of mice supplemented with the probiotics. Animals from 21, 28, 35, 42, 54, 61, and 180 days old, received *L. casei* CRL 431or *L. paracasei* CNCM I-1518, upon 7 and 5 days, respectively. Weaning mice (21 days old) that immediately received the supplementation with the probiotic Lc 341 or Lp1518 bacteria in their diet, for 5 or 7 days, did not show an antimicrobial activity in their intestinal fluids. In contrast, at 35 days old, an important reduction with respect to control animals, in the CFU number of *S. aureus*, began to manifest (18.75 ± 1.46, 20.75 ± 3.46, and 29.25 ± 3.75 × 10^9^ CFU/ml, Mean ± SEM, for Lc 431, Lp 1518 and Conventional diet, respectively), to became significant at 42, 54, and 61 days old mice. Most importantly, this antibacterial activity continued slightly high in aging mice (180 days old mice) that received the supplementation with the probiotics lactic bacteria (**Figure 6**).

**FIGURE 7:**
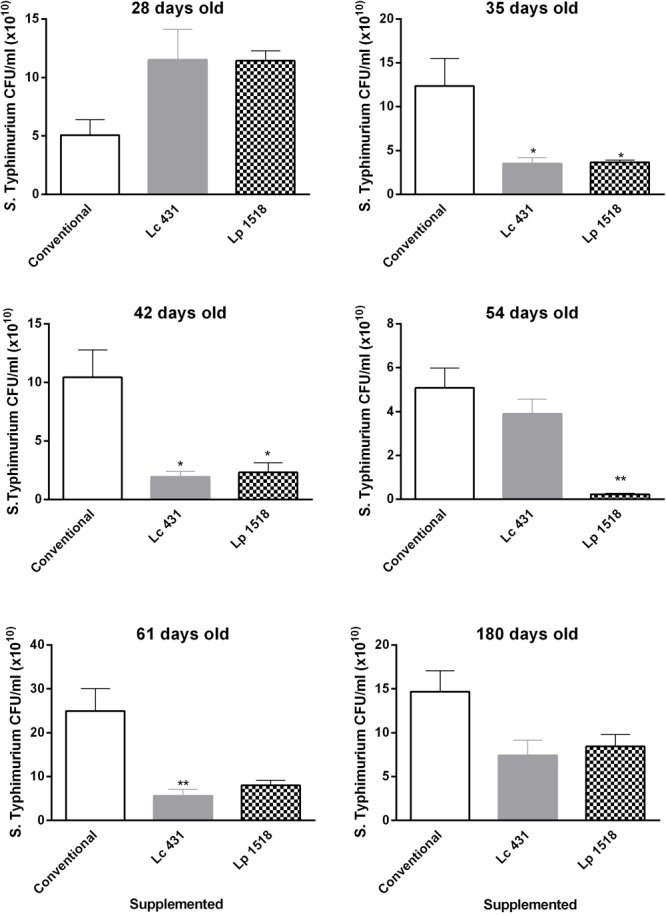
Antimicrobial activity of the intestinal fluids against pathogens, throughout the lives of the animals. Mice of 21, 28, 35, 42, 54, 61, and 180 days old were fed with Conventional diet, *L. casei* CRL431 or *L. paracasei* CNCM-I 1518, for 7 and 5 days, respectively. At this time samples of intestinal fluids were taken and studied for their *S.* Typhimurium antimicrobial activity after 2 h of incubation by plate count agar. Results were expressed as CFU/ml (mean ± SEM). Results were expressed as the differences in the CFU/ml before and after the incubation of the bacteria with the intestinal fluids. The statistic differences were calculated with respect to the control group that received conventional diet ^∗^*p* < 0.05, ^∗∗^*p* < 0.01, ^∗∗∗^*p* < 0.001.

Similar results were observed for *Salmonella* Typhimurium. Thirty five day mice that were previously fed for 7 or 5 days with Lc 431 or Lp 1518, respectively, showed a significant antimicrobial activity against *Salmonella*, with respect to animals that received conventional diet (*p* < 0.05). Oral supplementation with Lp 1518 increased between 3 to 10 times the Salmonella microbial activity of the intestinal fluids of 35, 42, 54, and 61 days old mice, compared with those observed in control mice (**Figure 7**). Besides, a tendency to diminish the CFU *Salmonella* count was observed in the presence of the intestinal fluids of 180 days old mice supplemented with both probiotics (**Figure 7**).

We then analyzed the antimicrobial activity of the intestinal fluids throughout the lives of the mice, against the same probiotics Lc 431 and Lp 1518. Despite the differences were not significant, the number of these microorganisms CFU were lower after exposition of *L. casei* CRL 431 and *L. paracasei* CNCM I-1518 to the intestinal fluids of 35, 42, 54, 61, and also 180 days old mice supplemented for 7 or 5 days with the probiotics, with respect to those control mice (**Table 1**). All together, these results show the relevance of the oral administration of probiotic bacteria as a tool to deal the growth of pathogens microorganisms and at the same time avoid the overgrowth of the probiotics, from early to old age.

**Table 1:** Intestinal fluid’s antibacterial activity against non-pathogen microorganism over the age, in mice fed with different diet.

Feeding	Microbial activity of intestinal fluids against		Mice days old
	*Lactobacillus casei* CRL431 (CFU/ml × 10^8^)	*Lactobacillus paracasei* CNCM-I 1518 (CFU/ml × 10^8^)	
Conventional	–2.12 ± 0.07	–0.08 ± 0.67	28
	6.25 ± 1.13	–0.81 ± 0.35	35
	11.50 ± 2.88	5.5 ± 1.15	42
	12.83 ± 3.062	–1.43 ± 0.23	54
	18.40 ± 0.03	18.33 ± 8.6	61
	0.21 ± 0.59	–1.83 ± 0.25	180
Lc 431	2.38 ± 0.55	0.49 ± 1.27	28
	4.2 ± 0.69	0.67 ± 0.71	35
	1.17 ± 0.42	4.00 ± 2.02	42
	1.60 ± 0.86*	–1.75 ± 0.08	54
	16.03 ± 0.32*	15.27 ± 7.43	61
	–1.07 ± 0.27	–2.20 ± 0.11	180
Lp 1518	0.94 ± 0.46	–0.57 ± 0.67	28
	3.86 ± 0.73	0.20 ± 0.55	35
	3.83 ± 1.12	–0.83 ± 0.21**	42
	1.66 ± 1.53**	–2.07 ± 0.18	54
	11.50 ± 1.89**	12.83 ± 0.91	61
	–1.62 ± 0.23*	–2.33 ± 0.03	180

*Results were expressed as the differences in the CFU/ml before and after the incubation of the bacteria with the intestinal fluids. Statistic differences were calculated with respect to the control group that received conventional diet ^∗^p < 0.05, ^∗∗^p < 0.01*.

### Oral Administration of Probiotics Does Not Modify Large Intestine Microbiota

**FIGURE 8:**
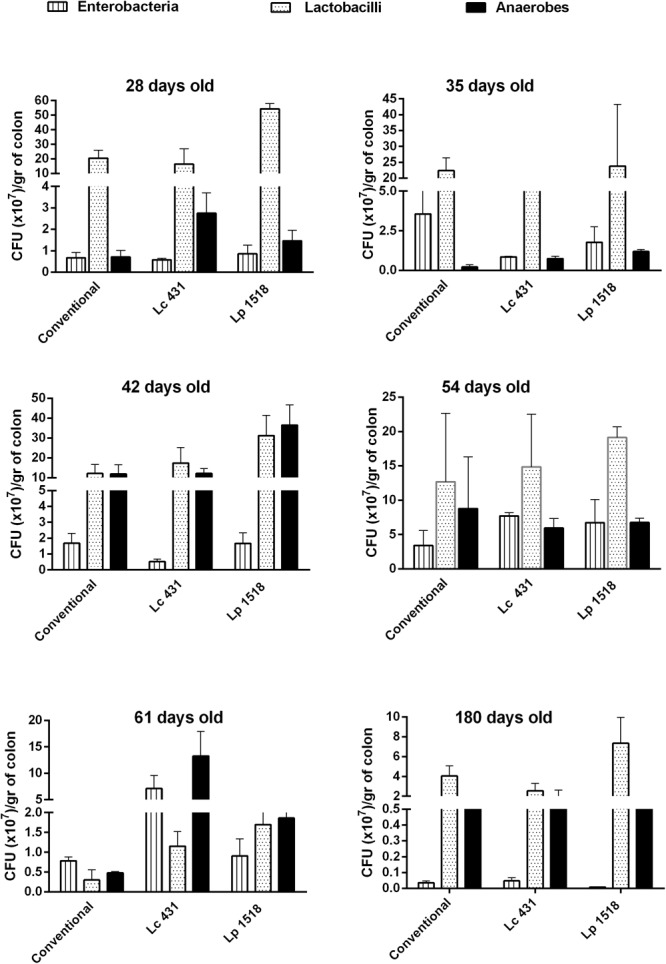
Microbial population present in the large intestine. Mice of 21, 28, 35, 42, 54, 61, and 180 days old were fed with: Conventional diet, *L. casei* CRL431 and *L. paracasei* CNCM-I 1518, for 7 and 5 days, respectively. At the end of this time, samples of large intestine were collected and total anaerobic bacteria, lactobacilli, and enterobacteria population were analyzed by plate count agar. Results were expressed as CFU/ml per gram of large intestine (mean ± SEM). Three independent experiments were performed.

With the aim of further characterize the role of Paneth cell stimulation in the mucosal immune system, large intestine microbiota was analyzed. Samples of large intestine belonging to mice from diverse ages (21–180 days old) fed with the Lc 431 or Lp 1518 for 7 and 5 days, respectively, were removed, homogenized, and placed in agar plate medium specific for different bacteria. We did not observe significant differences in the count of total anaerobic bacteria, lactobacilli, and enterobacteria at any of the times analyzed. Although a slight increase was observed at 42 days in the anaerobic bacteria in Lp 1518 fed mice, the differences were not statistically significant (**Figure 8**). These results demonstrated that probiotics did not significantly modify the large intestinal microbiota population analyzed.

## Discussion

The widespread use of conventional antibiotics has contributed to a huge increase in the number of resistant bacteria. Accordingly, AMPs emerge as an interesting alternative therapy that combines both their antimicrobial and immunomodulatory properties in the same molecule. Several studies both in the laboratory and in the clinic, confirm that emergence of resistance against AMPs is less probable than observed for conventional antibiotics, and provides the impetus to develop AMPs as sentinels to fight pathogens (Kang et al., 2014).

The relevance of AMPs can be illustrated in the fact that these molecules provide plants, fungi, and invertebrates of a robust and crucial defense against microbes without the help of lymphocytes, thymus, or antibodies acquired immunity (Zasloff, 2007). Upon exposure to viable or heat-killed bacteria or to microbial products such as lipopolysaccharide and lipoteichoic acid, Paneth cells release their granules, resulting in increased concentrations of antimicrobials in the intestinal lumen (Porter et al., 2002).

Several AMPs are being developed by the pharmaceutical industry, some of them even have reached preclinical or Phase II studies (Zasloff, 2002). Anyway, the high cost related to the production of these molecules under good manufacturing process (GMP) must not be underestimated.

The present study endorses the use of *L. casei* CRL 431 and *L. paracasei* CNCM I-1518 as a safe, effective and cost-effective tool to increase *in vivo* antimicrobial intestinal activity of Paneth cells. Previously, it has been demonstrated that lactic acid bacteria expose their probiotic effects in a dose-dependent manner, being able to kill pathogens, inhibit probably adhesion and invasion of pathogens, inactivate toxins and compete for limited resources successfully (Galdeano et al., 2007; Oelschlaeger, 2010; Johnson and Klaenhammer, 2014; Aktas et al., 2015; Dwivedi et al., 2016). Probiotic bacteria such as *Escherichia coli* Nissle 1917 and some lactobacilli have been shown to induce AMPs strongly (Wehkamp et al., 2004). *E. coli* Nissle 1917 (Mutaflor) is the oldest known (in use since World War I, 1917). Its efficacy in maintaining remission in Ulcerative Colitis (UC) has been shown in different placebo-controlled double-blinded studies, and this strain is now recommended as a guideline therapy by the Deutsche Gesellschaft für Gastroenterologie (Kruis et al., 2004).

In this study, we clearly demonstrated by histological analysis of intestinal tissue, that mice fed with both probiotic bacteria Lc 431 or Lp 1518 increased the number of the Paneth cells at the base of small intestinal Lieberkühn crypt (**Figures 1, 2**). Eosin intensely stains the basic granules in vertebrates. It has been well documented that the basic content in Paneth cells corresponds to molecules with antimicrobial activity (Porter et al., 2002). Considering this, we then analyzed the antimicrobial activity present in the intestinal fluids in 10 mM sodium phosphate buffer at pH 7.4, based in previous published results. Porter et al. (1997), assayed the microbicidal activity of recombinant human intestinal defensin 5 (rHD-5) in the presence of different salt concentration and a varied of pH (5.5 to pH 8.5). The authors observed that for wild-type *S.* Typhimurium, the activity of rHD-5 at pH 8.5 did not differ from the activity observed at pH 7.4. In contrast, at pH 5.5 defensin activity was reduced, even though bactericidal action was still detectable. Paneth cells are located in the small intestine, where the pH changes from pH 5 to pH 8 from the proximal to the distal end. These changes in pH could affect the activity of AMPs by modifying either the peptide or its target.

We observed a strong decrease in the CFU/ml of *S.* Typhimurium and *S. aureus* by plate count agar, after the incubation of the pathogens in presence of the intestinal fluids of mice fed with both Lc 431 and Lp 1518 (**Figure 3**). These results suggested that intestinal antimicrobial activity (defined by us as a change in the growth kinetic of the pathogen or the probiotics bacteria) can be stimulated *in vivo* by oral probiotics to display a potent killing effect. Anyway, it cannot be discarded that the metabolites produced upon the probiotic ingestion or the intestinal microbiota itself, could also be contributing to the observed effect.

Many hypotheses have been proposed about the mechanism of action of AMPs. The widespread known, based on their cationic and amphipathic nature, resides in the peptides interacting with cytoplasmic membrane, fatal depolarization and creation of physical holes that cause leakage of cytoplasmic content and widespread cell fragmentation (Zhang et al., 2010; Mukherjee et al., 2014). Accordingly, we observed strong damage in *S.* Typhimurium and *S. aureus* pathogens with clear loss of cell membrane integrity and cell wall fragmentation (**Figure 4**) after exposition them with the intestinal fluids of mice fed with Lc 431 and Lp 1518, by electron microscopy. By contrast, no damage of bacteria was observed when they were incubated in the presence of medium with low concentration of salts (control). These findings confirm AMPs release by Paneth cell as an active barrier to kill microorganisms, reduce translocation of pathogens across mucosal and prevent enteric infection. Moreover, the destruction of microorganisms by AMPs would be the link between the microbicidal activity and the stimulation of the immune response attributed to AMPs. The generation of pathogen-associated molecular patterns (PAMPs), such as cell wall degradation products or unmethylated microbial DNA, would promote the recruitment of innate immunity masters and APC activation (Rivas-Santiago et al., 2015) as well as amplify even more the release of inducible AMPs by Paneth cells. The effective epithelial responses to bacterial infection would prevent or reduce the frequency of focal neutrophilic infiltration and crypt abscess and minimize the establishment of potential portals of entry for microbes. Our results suggest the increase in the antibacterial activity of the intestinal fluids of mice, would be others of the mechanism by which probiotics protects against *Salmonella* infection. In that regards, we have previously demonstrated that *L. casei* CRL 431 increase animal survival, decrease pathogen spreading outside the intestine and local inflammatory response (de LeBlanc Ade et al., 2010; Castillo et al., 2011, 2013).

We also analyzed the antibacterial activity against the same probiotics bacteria. Interestingly, we observed fewer recounts of Lc 431 and Lp 1518 when those bacteria where incubated with the intestinal fluids of animals supplemented with both probiotics, in comparison to conventional diet (**Figure 5**). These results suggest that the growth rate of the bacteria was lower in the presence of intestinal fluids of mice fed with the probiotics than those obtained upon conventional diet. This antimicrobial activity was also revealed by the presence of bacteria fragments by electron microscopy. These fragments could be able to internalize across the intestinal epithelial cells, contact and mediate the activation of the intestinal mucosal immune system elicited by probiotic lactic bacteria. We have previously observed that probiotic bacteria were present in the lumen of the intestine or in the apical surface of the epithelial cells, but inside the intestine cells there were only antigenic particles from bacteria, probably products of the intestinal enzyme degradation. These results suggest that the whole bacteria cannot be introduced through the intestine cells and that only the degradation product of the bacteria are able to make contact with the immune cells (Galdeano and Perdigón, 2004). This fact allows the maintenance of the integrity of the epithelial barrier, in contrast with pathogens, which are able to invade the intestinal tissue. Moreover, the fast clearance of these particulate antigens from the gut would allow the regular consumption of probiotics without adverse effect to the host.

A pending challenge for immunologists is how to achieve an efficient stimulation of the immune system in elder people. With this in mind we analyzed whether probiotics can strengthen intestinal barrier by increasing antimicrobial activity in the intestinal fluids throughout the lives of the mice. Importantly, 52 and 61 day old animals supplemented for 5 and 7 days with *L. casei* CRL 431 and *L. paracasei* CNCM I-1518, respectively, displayed a clear reduction in the number of both pathogens studied, *S. aureus* and *S*. Typhimurium (**Figures 6, 7**). Oral administration of probiotics increased the intestinal antimicrobial activity when administered in young mice (35–42 days old) but also in elder animals, providing an effective shield against Gram (+) and Gram (-) pathogens.

In this sense, the role of Paneth cells and the intestinal AMPs to avoid intestinal bacterial translocation was firstly reported by Salzman et al. (2003) in an HD-5 transgenic mice. Besides, Vaishnava et al. (2008) demonstrated that the expression of MyD88 in Paneth cells is sufficient to limit *Salmonella* penetration across the mucosal barrier and systemic dissemination. Later on, Teltschik et al. (2012) associated the lack of Paneth cell function, especially α-defensins HD5 and HD6 to decrease mucosal microorganism killing, bacterial overgrown, translocation and systemic inflammation. Moreover, the slightly antimicrobial activity observed in **Table 1**, is really interestingly because it allows that probiotics can be use as adjuvants of the immune response at different ages, without the risk of an overgrown lactic acid bacteria.

In the intestinal lumen, commensal bacteria colonize and elicit beneficial effects in the host. That requires a complex and fine-tuned function of AMPs involved not only in removing pathogens but also in establishing symbiosis with the normal intestinal microbiota (Nakamura et al., 2016). After probiotic oral administration we did not observe with respect to animals fed with a conventional diet, differences in the number of total population of enterobacteria, anaerobes and lactobacillus in the large intestine (**Figure 8**). However, an extensive study of the large intestine microbiota composition should be performed by sequencing the V4 region of bacterial 16S ribosomal RNA gene, a revolutionary and useful technique to detect diversity and abundance of the microbiome.

Importantly, our results suggest probiotics were able to increase the killing of the pathogens without modify the intestinal homeostasis at any of times in which they were administered. The disruption of the critical balance between AMPs and luminal bacteria explains gastrointestinal infections and diseases (Wehkamp et al., 2009). An interesting point will be to identify the AMPs involved in this result. Masuda et al. (2011) demonstrated oxidized α-defensin cryptdin-4 (Crp4) has only minimal or no bactericidal activity against commensal bacterial species. By contrast reduced r-Crp4 had potent bactericidal activities against both commensals and non-commensals. *In vivo* degradation and inactivation of r-Crp4 before secretion should be an important mechanism to prevent the perturbation of small intestinal microbial homeostasis. Additionally, no RegIIIγ-dependent alterations in the luminal flora were evident in RegIIIγ-deficient mice suggesting that the antibacterial protein was inactivated, degraded, or sufficiently diluted when it reached the intestinal lumen (Nakajima et al., 2003).

The oral administration of probiotics will open a new avenue for the effective stimulation of Paneth cells and the intestinal antimicrobial activity. Oral probiotics can also be effective in a combined therapy to enhance the potency of existing antibiotics *in vivo*, reducing the doses required and so the appearance of antibiotics resistances. AMPs form an arsenal of weapons to combat microbial pathogens and regulate symbiosis between commensal bacteria and the host mucosa.

## Author Contributions

SC and CM-G carried out the microbiological work and the animal studies and wrote the draft of the manuscript. GP, RW, and JDP conceived of the study. SC, CM-G, and GP designed the experiments. SC performed the statistical analyses and prepared the figures. GP revised it for significant intellectual content. All authors read and approved the final version of the manuscript.

## Conflict of Interest Statement

The authors declare that the research was conducted in the absence of any commercial or financial relationships that could be construed as a potential conflict of interest.

## Footnotes

1 http://www.iibyt.conicet.unc.edu.ar/files/Reglamento-Formulario-CICUAL-IIByT.doc. Regulation and forum of the Institutional Committee for the Care and Use of Laboratory Animals (CICUAL) IIByT.

2 https://grants.nih.gov/grants/olaw/guide-for-the-care-and-use-of-laboratory-animals.pdf. Guide for the care and use of laboratory animal.
